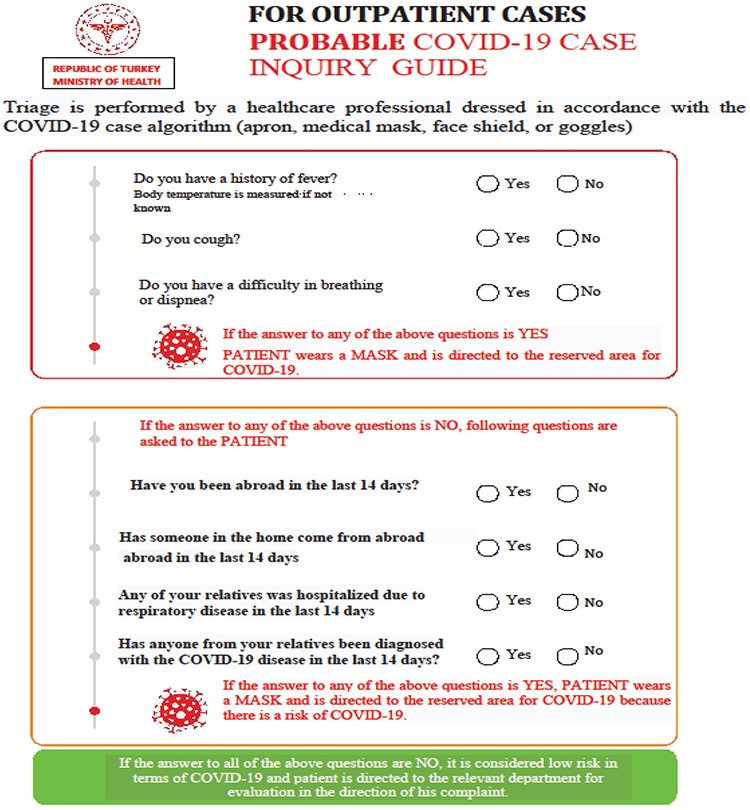# Guide for Nuclear Medicine Applications During the COVID-19 Outbreak

**DOI:** 10.4274/mirt.galenos.2020.33600

**Published:** 2020-04-29

**Authors:** Aslı Ayan, F. Suna Kıraç

**Affiliations:** 1 University of Health Sciences Turkey, Gülhane Training and Research Hospital, Clinic of Nuclear Medicine, Ankara, Turkey; 2 Chairman of TSNM Radiation Safety and Quality Control Working Group, İzmir, Turkey

**Keywords:** Coronavirus, COVID-19 outbreak, nuclear medicine staff, positron emission tomography/computerized tomography, infection protection rules, disinfection

## Abstract

A viral pneumonia rapidly spread from Wuhan, China to all countries in late 2019. In February 2020, WHO named as Coronavirus Disease 2019 (COVID-19) and declared the pandemic on March 11, 2020. To prevent the spread of COVID-19, Ministry of Health of Republic of Turkey and international institutions have published documents defining hygiene rules. After the lung computerized tomography (CT) findings which are important in the diagnosis of COVID-19 are described, protection measures against infection were defined in radiology departments. There is no publication involving protection measures for diagnostic and therapeutic procedures in nuclear medicine (NM) (appointment, patient acceptance, imaging and treatment procedures, disinfection etc). There are two reports on CT findings suggesting COVID-19 in ^18^F--fluorodeoxyglucose positron emission tomography/CT scan. These lung findings detected in hybrid images will be helpful in the early diagnosis of pulmonary involvement. Infected cases may be asymptomatic and can unintentionally disseminate the virus to surrounding people. This advisory guide has been prepared to avoid infection risk in NM clinics. During the COVID-19 outbreak, staff must use proper personal protective equipment and patients should be evaluated as the elective case according to clinical status. A questionnaire should be made for COVID-19. In cancer cases requiring urgent treatment, radionuclide treatment (RNT) should be planned according to the COVID-19 test result. If the result is negative, RNT can be applied; but if not or if the symptoms are present, RNT must be postponed. Following imaging procedures, scanners and room surfaces should be cleaned by personnel with proper disinfection training.

## Abstract (tr)

2019’un sonlarında, viral pnömoni Wuhan, Çin’den hızla tüm ülkelere yayıldı. Şubat 2020’de Dünya Sağlık Örgütü Coronavirus Disease 2019 (COVID-19) olarak adlandırdı ve 11 Mart 2020’de pandemi geliştiğini açıkladı. COVID-19 enfeksiyonunun yayılmasını önlemek için, TC. Sağlık Bakanlığı ve uluslararası kurumlar hijyen kurallarını tanımlayan broşürler yayınladı. Bilgisayarlı tomografi (BT) görüntülerinde COVID-19 tanısında önemli olan akciğer bulgularının tanımlanmasıyla radyoloji bölümlerinde enfeksiyondan korunma önlemleri açıklanmıştır. Nükleer tıpta tanı ve tedavi işlemleri için (randevu vermek, hasta kabulü, görüntüleme ve tedavi işlemleri, dezenfeksiyon gibi) korunma önlemlerini içeren yayın yoktur. ^18^F-florodeoksiglukoz pozitron emisyon tomografi/BT taramada COVID-19’u düşündüren BT bulguları hakkında iki yayın vardır. Hibrid görüntülerde saptanan bu akciğer bulguları akciğer tutulumunun erken tanısında faydalı olacaktır. Enfekte olgular asemptomatik olabilir ve farkında olmadan virüsü çevresindeki kişilere yayabilir. Öneri niteliğindeki bu kılavuz Nükleer Tıp kliniklerinde enfeksiyon riskini önlemek amacı ile hazırlanmıştır. COVID-19 salgını sırasında, personel uygun kişisel koruyucu ekipman kullanmalı ve hastalar klinik durumlarına göre elektif olgu olarak değerlendirilmelidir. COVID-19 açısından anket yapılmalıdır. Acil tedavi gereken kanser olgularında, COVID-19 test sonucuna göre radyonüklit tedavi (RNT) planlanmalıdır. Sonuç negatif ise RNT uygulanabilir. Sonuç pozitif ise veya semptomlar varsa, RNT ertelenmelidir. Görüntüleme işlemlerinden sonra, tarayıcılar ve oda yüzeyleri dezenfeksiyon konusunda uygun eğitim almış personel tarafından temizlenmelidir.

## Introduction

In late 2019, a new coronavirus was isolated from a group of patients admitting with viral pneumonia in Wuhan, China. With the increasing global people movement, the major concern over the possibility of this novel virus spreading and reaching pandemic levels has unfortunately now come true. In the first group of patients, the virus was believed to be transmitted from the wet “seafood market” in Wuhan. Since then, numerous reports which are related with human-to-human transmission have been published. It has rapidly spread to all countries world-wide. New countries affected by the virus are reported every day.  The novel virus is one member of the coronavirus family and Coronavirus Disease 2019 (COVID-19) spreads from human-to-human like flu (1).

Due to the increasing number of patients in our country since Mid-March 2020, more attention has been given to infection control measures especially in hospitals that have the potential to spread the virus not only among patients but also healthcare personnel. These measures should be implemented at the national level, not just at the hospital level and regional. It is important to comply with the general hygiene rules for public persons and health professionals. After the World Health Organization (WHO) declared that the COVID-19 global pandemic has developed on March 11, 2020, general hygiene rules have been published by Republic of Turkey Ministry of Health and international related institutions to prevent the spread of disease and to avoid new infected cases (Annex 1) (2). However, more strict and branch-specific protection rules are necessary, since healthcare professionals are more likely to come in contact with the infected and/or probable infected cases. Because the lung findings in the computerized tomography (CT) images are useful in the diagnosis of COVID-19, although there are articles about how they can be careful to reduce to the risk of outbreaks in the radiology departments, there are very few application recommendations for nuclear medicine (NM) clinics (1,3,4). Despite the similarities, there are pertinent differences between radiology and NM regarding the urgency of diagnostic and therapeutic applications, the length of patient contact, the ability for portable imaging instruments and the duration of scans, which necessitate a separate set of advice. Therefore, just like infection protection algoritms prepared by other medical specialities, preventive applications guide has become mandatory in the NM clinics, too.

NM departments are the clinics that necessarily apply the principles of infection protection published by WHO in their daily practices due to the ionizing radiation protection measures (distance, time, shielding). Similar to the radiology departments, NM specialists, nurses and technicians are generally at the highest risk of exposure to the COVID-19. COVID-19 is believed to be mainly transmitted through respiratory droplets and close contact with infected patients or with the asymptomatic carriers in the incubation period. Also, it may be transmitted indirectly through touching in contaminated surfaces or objects. There have been no reports of airborne transmission of the COVID-19 virus to date. However, it can not be denied that some aerosol-forming process such as supportive oxygen therapy or intubation, would be able to cause transmission in the healthcare units. There are some experiences providing important recommendations and measures, which have been vested from prior coronavirus epidemics of SARS and MERS outbreaks which in the same class with COVID-19, to combat the current outbreak (1,3).

Nuclear medicine department is partially lucky compared to other branches because the majority of imaging methods and radionuclide treatment (RNT) performed are elective and tend to be outpatient. In addition, NM workers will have the chance to take the necessary precautions, since the new studies will usually be performed in the patients who have already been hospitalized and secreened for COVID-19. Nevertheless, the absence of portable single photon emission computerized tomography (SPECT) or positron emission tomography (PET)/CT instruments is the most important risk. Setting appointments over the phone will help to identify infected and probable patients, it partially will prevent COVID-19 contamination to the patients and staff, and also the development of outbreak in NM clinics. However, the risk of encountering the virus will not be zero by patients who have not been diagnosed yet, with no suspicious history in terms of disease, but any form of asymptomatic infection at the time of admission. Therefore, this guideline including some suggestions has been prepared with a goal for providing the protection of patients and healthcare workers in the NM clinics that continue to perform the diagnostic and therapeutic procedures even under the risk of contamination by COVID-19 virus which is defined as pandemic by WHO.

The recommendations set out in this guide should be adapted to the clinics’ own working conditions, fields of activity and the number of patients.

## Patient Registration

1.  In the patients whose new appointments will be arranged, the clinician or assisstant physician responsible for the patient referring to the NM clinic should be asked to give detailed information on COVID-19 and the patients’s previous illnesses.

2.  During recourse and enrollment to the department, the patients travelling to the country with known COVID-19 outbreak should be encouraged to self-report, especially when they are symptomatic (Annex 2) (2).

a.  The first registration, if possible, should first be accepted by phone and an inquiry should be made in this regard.

b.  If it is not possible to make an appoinment by phone, the necessity to fill the notification form prepared for patients to declare their own information in the NM secretariat will be useful in the identifying infected and suspicious cases (Annex 2).

3.  During admission, the fever measurement of the patients who registered for outpatient-treatment, and who will undergo a diagnostic procedure may be useful in distinguishing asymptomatic patients. In the large departments dealing with outpatient or referral service to the healthcare provider, thermal screening by thermal scanning and mass screening systems that measure the skin temperature at high-speed using temperature measurement equipment as used in airports must be considered.

4.  Healthcare staff should well know the symptoms of COVID-19 infection such as fever, dry cough, fatique and shortness of breath. Additionally, they should be aware that patients are asymptomatic carriers of the virus and that history of contact with a suspicious case is important for probable infection.

5.  If there is a suspicious case for COVID-19 when setting an appoinment over the phone, the responsible NM specialist should be informed by the interviewing staff. After that, the competent authority must be notified immediately and the patient should be transferred to the competent hospital.

6.  Advisory staff who are arranging the patient procedures in nuclear medicine, making an appoinment and providing the necessary documents exchange should be properly trained for the risk of COVID-19 transmission. It is strongly recommended to use anti-virus protective equipments such as disposable head caps, disposable masks, disposable gloves and hospital-only work clothes.

7.  When such patients are identified, they should be placed in a separate waiting area and must be consulted to the infectious diseases specialist or local line accross, our country 184, should be called in the case of strong suspect.

8.  Taking into consideration of current information, the patients should use a surgical mask in order to minimize the potential risk of transmission while waiting for results of assessments.

9.  If possible, it is recommended to postpone all patient procedures (for diagnostic or therapeutic purposes) until the results of the COVID-19 tests are obtained.

10.  When the COVID-19 endemia develops, giving disposable masks to all patients to put on while they are in the department should be considered.  This practice must be applied at the expense of the patient’s objection.

11.  If there is more than one receptionist, it is recommended that the work desks are placed at a distance of 1 m, and similarly, a barrier may be created in front of the counseling desk with a distance of 1 m between the desk and the patients or accompanying persons.

## Registration Reception Hall Waiting Area

1. Waiting areas must be close to hand washing facilities, masks should be in easy reach. Patients can be encouraged/provided to follow basic hygiene practices.

2. Since the risk of coronavirus transmission increases within one (1) m, the waiting area should have enough space in order to provide enough distance while waiting. The concept of sufficient distance should be designed according to the daily patient load of each clinic. In NM clinics that cannot provide 1 m distance measure between the patients, keeping the admitted patient and/or accompanying persons outside the clinic for providing the social isolation distance; or that to take in the patients individually and in a way that will not cause a confluence in the waiting area, or limiting the number of new appointments based on the size of the waiting area is recommended.

3. The reception hall and waiting area should be good ventilated by equipment with a high-efficiency particulate air filter.

## If the Patient has been Called for Procedure

1. NM staff welcoming patients such as NM technicians or nurses are individuals who will be in the most close contact with infected patients.

2. Because physical contact is unavoidable during catheter insertion intravenously and it may take a long time, it is very important to identify probably infected patients before this step.

3. Therefore the informations in terms of COVID-19 infection should be added into the cases’ anamnesis (Annex 2).

4. If COVID-19 outbreak is defined in the country, it is mandatory that workers use proper personal protective equipment (PPE). PPE (level 1 protective equipment) is generally sufficient in the NM clinics. Level 1 protective equipment includes a disposable surgical mask, special work clothes, disposable latex gloves and/or disposable insulating clothing. Additionally, staff wearing contact lenses must switch to glasses to protect health. Wearing eyeglasses can shield their eyes from probable transmission by contaminated hands or infected respiratory droplets (5). However, level 2 PPE should be used during the examination and imaging of the confirmed or probable patient, and during cleaning of the equipment used to the patient with a suspicious or confirmed disease. Unlike level 1, level 2 PPE requires the use of N95 or equivalent mask, disposable protective suit, googles or face shield (3). Especially the beard and/or mustache interfere with the N95 masks or respirators fitting to entire face and thereby virus transmission risk increases. It is important to be clean shaved for complete protection against infection (6).

5. However, the procedures performed with full PPE are difficult and may affect the ability of personnel to handle the same patient load as before.

6. These practices may change depending on the number of positive cases in the hospital and the departmental success to catch infected or suspicious patients who have been admitted to the department for any procedure or the number of confirmed or probable COVID-19 cases referred.

## Recommendations for The Waiting Rooms of Radioactive Patients

Most of NM imaging and procedures require a processing time from several minutes to several hours following radiopharmaceutical administration. During this time,

1. 1 m distance between patients placed in the separate radioactive patient areas (PET patients) or in the common waiting rooms (for gamma camera patients) should be maintained to prevent novel coronavirus transmission among patients.

2. Waiting rooms of radioactive patients should be good ventilated by an equipment with a high-efficiency particulate air filter.

3. The crowded rooms may increase possible transmission risk due to cases who are asymptomatic and have an inappropriate history. It is adviced to limit the number of patients according to the size of radioactive patient room.

4. Similar measures as those defined for the non-radioactive patients and accompanying persons in the registration hall should be applied to all other stages after radiopharmaceutical administration. First of all, all of them should be supported to wear a protective mask.

## When the Patient is Scanned

1. Once patients’ scan is complete, scanners and room surfaces should be disinfected to avoid potential spread.

2. As a general rule of Public Health Units;

a. In the absence of visible pollution after scanning, disinfectants produced for especially the hospitals or the 1/1000 (1000 parts per million) parts of chlorine solutions containing are recommended to use in the disinfection of scanning instruments and clinical rooms used (7).

b. Terminal cleaning procedures must be applied in case of the presence of patient secretions, urine or stool contamination (7).

c. Adequate and appropriate training is recommended for cleaning staff to work properly.

## The Safety of Nuclear Medicine Staff

1. The same precautions and tests recommended to the patients at the time of admission should also be valid for NM personnel (eg technicians, nurses, NM specialists) who are in close contact with the patient.

2. Personnel who is in contact with patients must wear a whole apron, gloves, surgical mask, protective eye glasses and shoe covers.

3. Personnel should be trained in the use of these equipments and be warned to correct their mistakes seen during usage.

4. Similar measures such as isolation at home, should be taken for the staff who has symptoms like fatique, fever, cough, or a history of travelling in areas with more widespread local transmission of COVID-19 or a history of contact with travellers visiting infected areas or contact with confirmed cases, for reducing the risk of virus transmission.

5. It would be appropriate for senior clinician and/or supervisors to advice workers not come to work if they feel unwell.

6. Due to the possibility of the presence of asymptomatic infected personnel, employees can be prevented being together by adjusting rest time.

7. If COVID-19 spread increases, the larger NM departments, may consider dividing their staff into alternative working groups to reduce the risk of viral transmission among the healthcare providers causing department work to fail.

8. All staff should receive proper training to ensure maximum compliance for measures.

9. Clinical staff should stay under observation at home to monitor the daily symptoms if they have come into contact with a probable case in less than 1 meter distance without PPE or if they directly or indirectly exposed to the patient’s respiratory secretions. If test result of the suspicious infected case who contacted with, is negative, the observation is terminated. But, if test result is confirmed as positive, the observation at home should continue for 14 days. Staff who have symptoms should be managed in accordance with the applications for COVID-19 probable case according to the COVID-19 Disease Guide and the COVID-19 case flow-chart published by the Ministry of Health of Republic of Turkey.

10. An emergency and business continuity plan should be prepared.

## Recommendations for Diagnostic and Therapeutic Applications with Radionuclides During Outbreak

### A. Myocardial Perfusion Scintigraphy (MPS)

1. Taking into consideration asymptomatic patients, patient in a good clinical condition without any strict contraindication criterion, performing pharmacological stress test (especially with vasodilatator agents) shortens the procedure time instead of treadmill exercise test. This recommendation can keep safe the staff and also the patient from possible transmission.

2. Since the pharmacological stress test will be performed at a less distance than 1 m, particular attention should be paid to the use of PPE. If possible, it is recommended to wear a disposable clothing over the lead apron, and to properly disinfect the materials and the clothes used during the procedure. Additionally, patient must wear a protective mask during the entire procedure.

3. Cardiac arrhytmias, myocarditis, low ejection fraction and sudden death cases have been reported due to SARS-Cov-2 (COVID-19) infection (8). There is no published MPS scintigraphy study including COVID-19 cases, yet. Another important issue here is that hydroxychloroquine preparations (hydroxychloroquine is a drug known with strong antimalarial effect. However, today it is used in the treatment of autoimmune disease rather than malaria treatment) has been reported to be effective in COVID-19 treatment and even for prophylaxis, may cause similar cardiac findings. The side effects of hydroxychloroquine drugs are not dose dependent in each case and may also develop idiosyncratic side effects in individuals who are sensitive to 4-aminoquinoline compounds. Therefore, it should be taken into account that side effects of the drugs may be thought as the cardiac manifestations of the disease. Additionally, it may cause serious retinopathy and skin lesions (9,10,11). It is recommended to be used prophylactically with the decision of the expert and under the supervision of specialist in the healthcare workers at high risk of disease.

### B. Lung Perfusion Scintigraphy

The most common symptoms in COVID-19 patients are high fever, cough, myalgia and fatigue. Pneumonia involving multiple lobes, bilaterally has been reported in the published studies. The SARS-Cov-2 pneumonia resulting extensive inflammation and infiltration is localized particularly in the posterior and peripheral zones of the lungs (12,13) (Table 1). However, patients especially who are at risk for pulmonary embolism (PE) such as deep vein thrombosis, malignancy, and presence of immobilization may be considered as PE because of that typical complaints are not detected and no severe infiltration findings in lungs in the early stages.

PE can lead to non-specific findings such as arterial hypoxemia and hypocapnia in the patients. These findings are also seen in chronic obstructive pulmonary disease, lung cancer or pulmonary fibrosis besides PE. The pulmonary X-rays will be helpful in the differential diagnosis of non-PE diseases. But its sensitivity is very low (40%). Although the sensitivity (82%) and specificity (96%) of perfusion-only study is inferior to combined ventilation/ perfusion study, these values are still high in the diagnosis of pulmonary embolism.

1. In the clinics where there is no SPECT/CT equipment, patients suspicious for PE should be referred to lung CT scanning in terms of the possible COVID-19 pneumonia, even if their body temperature is not high. After that if necessary, pulmonary perfusion scintigraphy should be performed following CT. Planar or SPECT perfusion study should be evaluated together with clinical findings and simultaneous CT images (14,15,16).

2. If SPECT/CT camera is available, hybrid imaging have to be performed; careful examination in terms of ground- glass opacities and pneumonic infiltration in the CT slices obtained, especially posterior and peripheral, subpleural zones, will provide useful informations in the the early diagnosis.

3. Lung ventilation scintigraphy must not be performed if there are findings mimicking COVID-19 pneumonia such as non-segmental perfusion defects, non-wedge-shaped perfusion defects, resembling lung parenchymal disease.

a. There is a risk of transmission of all viral and bacterial infections, including COVID-19, by means of ventilation devices.

b. Additionally, the lung ventilation procedure can cause aerosolization and the formation of microdroplets. Therefore, lung ventilation study should not be performed in patients with probable PE during the pandemic (6).

c. If necessary, perfusion-only study should be settled in these cases as defined above.

4. Pneumonia and PE findings sometimes overlap. Diagnostic lung CT imaging should be performed in both conditions.

5. The flow-chart recommended in probable cases should be followed.

The Tc-99m macroagregated albumin (MAA) particles used for lung perfusion scintigraphy are 15-100 µm in size and the particle distribution accurately shows regional lung perfusion. Since MAA particles block pulmonary capillaries and precapillary arterioles, the number of particles injected is important. Normally 100.000-500.000 (ideally 400.000) particles are injected during perfusion study. However, 60.000 particles are sufficient for obtaining uniform distribution of activity reflecting regional perfusion. Taking into account that the number of pulmonary capillaries and precapillary arterioles present in the lungs, the administration of up to 400.000 MAA particles will result in obstruction in a very small fraction of pulmonary vessels. But it is recommended to inject the minimum number of MAA particles (60.000 particles) which sufficient for good quality images (14,15). In this way, developing widespread microemboli will be prevented and kept pulmonary functions in the patients with the possibility of COVID-19 pneumonia which can cause severe damage in the lungs.

### C. Hybrid Imagings

1. There is increasing number of reports which describe CT findings of COVID-19 associated pneumonia. Recently a manuscript containing 4 (four) cases about random CT findings suggesting COVID-19 in the ^18^F--fluorodeoxyglucose (FDG) PET/CT scans and a case report including a patient who underwent FDG PET/CT scan due to a lesion in the lung were published. COVID-19 was detected in one case in each publication (17,18,19).

2. If interlobular septal thickening and ground-glass density areas with high metabolic activity are detected in the cases who underwent to ^18^F- FDG PET/CT imaging for other reasons, these cases should be referred to the relevant institutions or departments.

3. Similar lung CT findings for COVID-19 pneumonia observed in the SPECT/CT slices of the thorax should be reported to the Infection Control Committee or to the call centers in the hospital by NM specialists.

4. Based on these cases, a more careful evaluation of PET/CT or SPECT/CT images of each patient, especially the lungs in the CT components, and reporting the lung findings observed by NM specialists will offer very useful informations to the clinicians in the early diagnosis of COVID-19 lung involvement (12,13). Because such patients may be asymptomatic, and being unaware, can spread the virus to the surrounding people and to the people who they are in contact with. Imaging findings are similar to previous coronaviruses such as MERS or SARS. These nonspecific findings become meaningful in terms of COVID-19 infection if they comply with suspicious case definition.

5. Information about the patient’s clinical history will be useful in the diagnosis for evaluation of radiology and NM images, as in routine practice. Risk factors such as chronic illness, the history of contact with confirmed patients and visiting to the places where COVID-19 infection spread should be investigated.

6. Staff performing injection and imaging of these patients must be questioned in detail for a history of contact with patient (such as duration, proximity, whether using PPE).

Table 1 shows the CT findings and their distributions regarding to lung lobes that can be observed during COVID-19 infection.

### D. Radionuclide Treatments

Although cancer patients have a higher risk than healthy people, there is still no published chronic disease and algorithm for COVID-19 infection in these patients. It is accepted that the presence of other being older than 65 years will rise the infection risk. Although there is a small number of reports on surgical treatments in cancer cases suspicious for COVID-19 infection or infected (20,21), any published report regarding RNT is not present, yet. If necessary, the guide for surgery of these cases may be applied. According to these rules;

1. The patients referred for RNT, should be evaluated electively based on their clinical condition.

a. A questionnaire should be made for symptoms and signs of COVID-19 infection, and answers should be recorded (Annex 2).

b. It should be questioned whether there are any infected or suspicious cases at home or in their immediate vicinity. It should be recommended to postpone the appointment and refer to the relevant institution or unit for the diagnosis of COVID-19, if there is a suspicion for coronavirus infection.

2. If the patient preparation for I-131 treatment or whole body scanning is not urgent, treatment should be postponed and replacement treatment should be given for euthyroidism in hypothyroid patients who are in the preparation period. If urgent treatment is necessary, recombinant TSH (rTSH) can be preferred, instead of hypothyroidism in these cases.

3. Patients who had an appointment for RNT other than I-131 should also follow the recommendations defined for patients who will be arranged new appoinments.

4. For all RNT including I-131, in case of requiring urgent treatment, the questionnaire for COVID-19 infection should be applied at initial referral and must be recorded.

a. If there is fever (>37.3), a history of travel to the outbreak area or contact with an infected person, social isolation at home is recommended for fourteen days before treatment. At the end of this period, reevaluation should be done.

aa. Treatment can be planned for the patients who COVID-19 is negative or without having symptoms following 14 days of isolation.

aaa. The patients with positive test results for COVID-19 or who have symptoms should be directed to the authorized units for infection treatment at first. After COVID-19 treatment, re-evaluation should be made and a novel appointment for RNT have to be arranged.

5. A flow-chart similar to cancer cases should be followed in patients whose I-131 treatment is planned due to hyperthyroidism. Antithyroid treatment should continue until COVID-19 is identified as negative.

6. During treatment, COVID-19 positive and negative cases, and also personnel applying treatment should be followed the measures suggested for people at high transmission risk but no with cancer diagnosis, and hygiene rules (5,6,12).

7. Dose rate values  of the patients regarding COVID-19 test positive soon after RNT should be recorded by the Radiation Protection Officer at the time of hospital discharge and the epicrisis related to treatment process should be given (22,23).

8. In case of deterioration in the general condition of these patients (use of a ventilator, intubation, hemodialysis) or death, relevant national and international guidelines should be consulted for measures and practices to be taken in terms of radiation safety and infection transmission (22,23,24,25,26,27,28).

### E. Disinfection Procedures in Nuclear Medicine Departments

1. Visual materials such as brochures and in-hospital broadcasts may be used to promote hand washing and good respiratory hygiene measures in the department.

2. All gamma camera gantries and image monitoring station mouses and keyboards, sphygmomanometer cuffs, all surfaces (tables, seats, chairs and beds), should be wiped with disinfectant regularly and after each contact with suspicious patients.

3. Make sure disinfectant bottles in the work area are easy accesible and refilled regularly.

4. The risk encountered at the time of cleaning is not the same as contacting an infected patient who coughs or sneezes. However, cleaning personnel who makes clean all areas in the department during work and out of working hours should be specifically trained for professional cleaning of potentially contaminated surfaces after high-risk patient contact (3,7). During cleaning time, cleaning personnel should;

a. Use proper PPE. Informations should be given on how to put on and take off this equipment and practical training should be done.

b. Informed about not touching their faces, especially mouth, nose and eyes.

c. Wear water-proof disposable gloves and use a surgical mask, eye protective or face shield, shoes cover.

d. Wear glasses instead of lenses (5).

e. Use alcohol-based hand disinfectants before and after wearing their gloves.

f. Use alcohol-based disinfectants with a virucidal effect before and after wearing surgical mask.

g. If there is a possibility of visible contamination with respiratory secretion or other body fluids; in addition to surgical masks and eye protection equipment, disposable protective clothing that covers the entire body should be worn prior to cleaning work.

## Take-home Messages

• For outpatient cases, the imaging process should be carried out in accordance with hospital policy. If imaging is really necessary, special patient waiting rooms equipped with high-efficiency particulate air filters are recommended.

• An algorithm must be developed to ensure that probable cases are identified in a timely manner.

• All staff must receive appropriate training to ensure maximum compliance measures.

• Staff should be contacted and given the message that they should stay at home even in the presence of mild COVID-19 symptoms.

• In case of signs of illness suggesting COVID-19 or confirmed infection in your personnel, emergency and business continuous workflow plans should be adapted in order to prevent the disruption of function.

## Conclusion

Although there are many issues raised for NM applications concerning the current COVID-19 outbreak, NM departments can make a significant contribution to reducing the effect of COVID-19 infection on patients and staff, if adequately prepared for PPE and disinfection procedures. The lessons learned from the current experience and the data obtained from the case groups will help to improve preparedness and address possible deficiencies in case of new outbreaks in the future.

## Figures and Tables

**Table 1:**
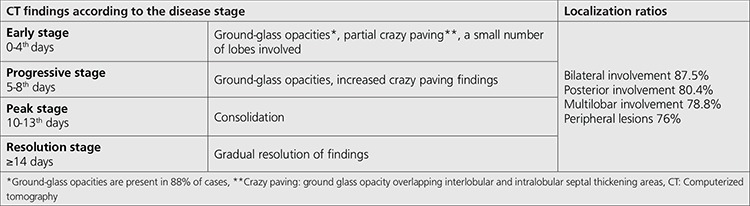
CT findings and their distributions in the lung lobes that can be observed during COVID-19 infection

**Annex 1:**
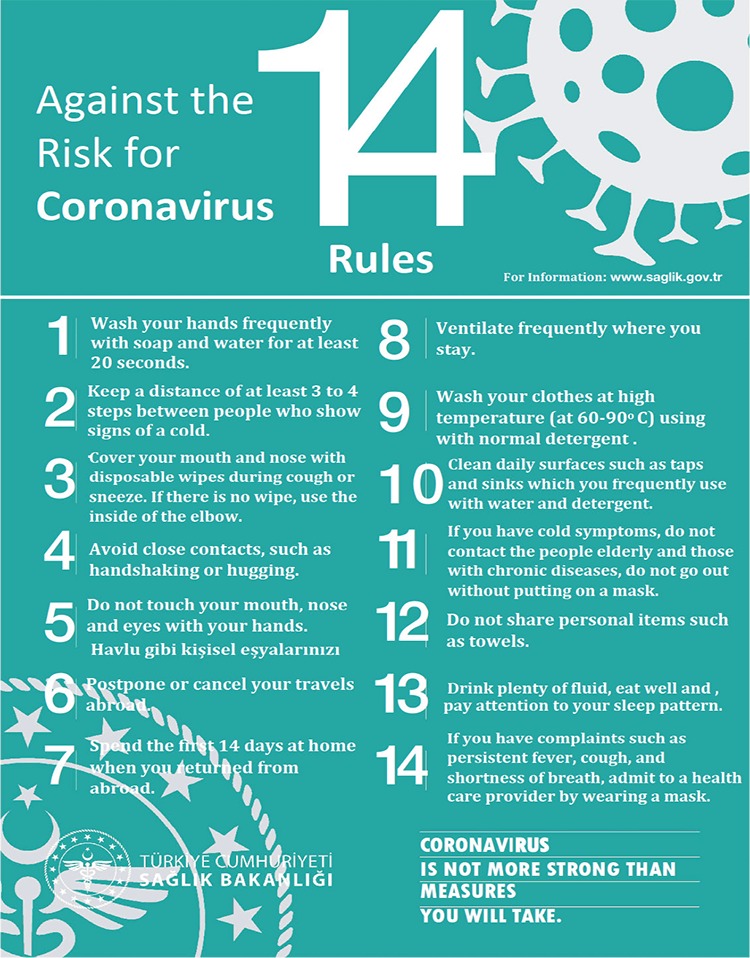


**Annex 2:**